# Potential deformation of the stented portion in partial frozen elephant trunk: a double-edged sword during retrograde flow under retrograde perfusion

**DOI:** 10.1186/s44215-026-00259-0

**Published:** 2026-04-20

**Authors:** Takeshi Shimamoto, Kenji Minatoya

**Affiliations:** Department of Cardiovascular Surgery, Kyoto University Hospital, Kyoto, Japan

**To the Editor:**

We read with great interest the case report by Niki and Esaki regarding unanticipated stenosis of the distal edge of a partial frozen elephant trunk (PFET) during retrograde perfusion via extracorporeal membrane oxygenation (ECMO) [1]. The authors highlighted that the non-stented “skirt” portion of the PFET is susceptible to severe narrowing under retrograde flow. As a group that has reported on the initial clinical outcomes of PFET [2], we wish to provide experimental insights that expand upon this observation.

The PFET was specifically designed to prevent distal stent graft-induced new entry. By reducing the number of stent wires from two to one and using thinner wires, the device successfully achieved a 43% reduction in spring-back force and a 29% reduction in radial force compared to conventional models [2]. While these modifications are highly effective in reducing stress on the aortic wall, they may also render the device more compliant under external pressure.

In our clinical series, we achieved a 100% technical success rate and zero in-hospital mortality [2]. However, we encountered one case with a significant pressure gradient between the upper and lower extremities postoperatively. To investigate this, we conducted a hydrodynamic simulation using a pulsatile flow model (37 °C, 120 mmHg, 50 bpm). The PFET was deployed into a dissected aortic model with a mock flap.

Our findings demonstrated that PFET narrowing occurs not only at the distal skirt but also within the stented portion during retrograde flow (Fig.1). Notably, while removing the 2-cm skirt improved the narrowing, significant deformation of the stented segment persisted. This suggests that the structural integrity of the PFET, optimized for lower radial force, is inherently sensitive to retrograde pressure gradients.

**Fig. 1:**
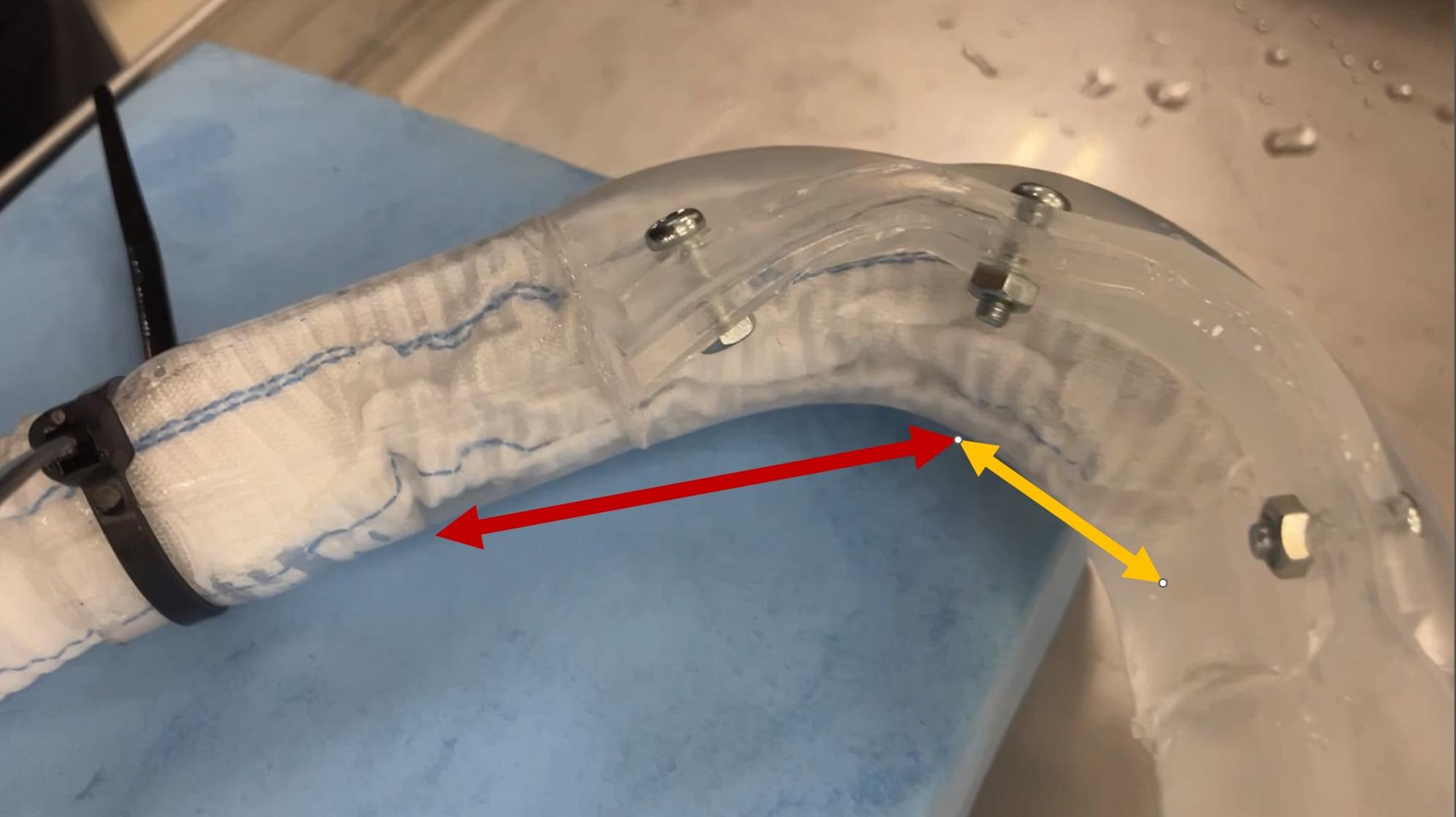
Hydrodynamic simulation of the partial frozen elephant trunk (PFET) under retrograde flow. The flow is directed from right to left, mimicking retrograde perfusion. Significant deformation is observed in both the distal skirt (yellow arrows) and the stented portion (red arrows)

PFET should be viewed as an advanced alternative to conventional unstented elephant trunks rather than a direct substitute for high-radial-force FETs. It offers superior handleability for second-stage surgery by avoiding “stent-graft-graft” anastomosis failure, where sutures and metal stents interfere to cause pseudoaneurysms [3]. However, surgeons must remain vigilant; when retrograde perfusion (e.g., ECMO) is required, the risk of collapse of both the skirt and the stented body must be considered to prevent malperfusion. Even conventional FET is susceptible to intraoperative malperfusion [4].

## Data Availability

The data and materials (such as experimental photographs) supporting the findings of this letter are available from the corresponding author upon reasonable request.
